# European Public Health News

**DOI:** 10.1093/eurpub/ckac005

**Published:** 2022-02-01

**Authors:** Dineke Zeegers Paget

**Affiliations:** EUPHA Executive director

Welcome to the first European Public Health News of 2022. In this section, you will find information on COVID-19 (again), fighting cancer and the upcoming European public health conference. Zeegers compares the COVID-19 pandemic with the AIDS pandemic. Despite many differences, there are general lessons to be learned. Smelov et al. from WHO Europe calls for using the windows of opportunity provided by COVID-19 to the urgent situation of fighting cancer. Finally, Busse combines our lessons learned from COVID-19 and the changes these lessons should bring to our health systems. This is the main theme of the 2022 Berlin conference where we hope to see you all sharing your work and networking with your peers.

## Our answer to pandemics—revisited

Zeegers PagetDineke
EUPHA Executive Director
[Aff ckab188.001-aff1]

I am writing this column on the 1^st^ of December—World AIDS Day—while we stand at the beginning of the 4^th^ wave of COVID-19. This World AIDS Day marks the 40^th^ year that AIDS has been identified as a specific illness and epidemic, infecting over 75.7 million people with HIV and killing over 32.7 million people from AIDS-related diseases. Over time, more and more treatments became available to manage HIV infection, slowly turning HIV infection into a chronic condition. In COVID-19 times, people living with HIV are more at risk as they have higher comorbidities and—sometimes—limited access to COVID-19 vaccines [Global HIV & AIDS statistics—Fact sheet | UNAIDS, last accessed 1 December 2021]. In comparison, COVID-19 was identified in December 2019 and—as of January 2022—the pandemic counted more than 292 million infections and 5.4 million deaths [COVID-19 pandemic—Wikipedia, last accessed 4 January 2022].

The HIV pandemic and the COVID-19 pandemic are very different especially in the transmission: HIV is transmitted through direct contact (body fluids) while COVID-19 is airborne. But both pandemics also have a lot in common, especially in the way legislators and politicians have reacted to both epidemics. In a 1997 study^1^, comparing AIDS-related legislation in the world, the author concluded that the reaction to pandemics has not changed that much and that—in future pandemics—similar reactions could be expected. And decades later, this is exactly what we see.

In general, legislators and politicians react to pandemics in three steps:

1. Denial that this epidemic/pandemic exist in their country/region;
2. Recognition that the disease is present; and
3. Measures to inform the public and take preventive measures as well as start an active search for a vaccine/treatment.

### The stage of denial

At the start of the AIDS epidemic, the problem was seen as an American and then Western world problem. It was only in 2003—more than 20 years later—that WHO’s Director General declared AIDS a global health emergency^2^. In the beginning, the only known ways of transmission were homosexual contacts and sharing of dirty needles among drug users, but it was quickly shown that health care workers were also at high risk (use of needles) as well as the whole population (heterosexual transmission). With the COVID-19 pandemic, things moved forward much faster. The virus was first found in Wuhan, China in December 2019 and WHO declared COVID-19 a global pandemic on 11 March 2020 (only 4 months later). However, countries were still denying that the virus existed in their country. While Italy was open about the seriousness of the infection, the US president at that time (Donald Trump) denied that there was a virus, something he is still communicating to his followers to this date. The differences in the timeline of the two pandemics is probably largely due to visibility. With AIDS, the biggest groups with a serious risk of infection were illegal behaving drug users and (sometimes illegal) homosexual behaviour. COVID-19 infects the whole population. Furthermore, AIDS happened in times where no internet and social media were available, whilst COVID-19 occurred in times with increased social media.

### The stage of recognition

In the stage of recognition, countries acknowledge the spread of the pandemic, but are still trying to contain it, meaning that measures are taken to restrict entry into the country. In the case of AIDS, there are reports of HIV-positive people being denied entry and send back to their country of origin, even when there was a World AIDS Congress organized in that country (1989, Canada). With COVID-19, the containment led to bans on entry from and on departure to certain countries (e.g. the UK had a ban on travelling from the UK in December 2020). AIDS remained a hidden disease for decades, because the groups affected the most were specific groups with high-risk behaviour. The blame game^3^ played a significant role in the AIDS pandemic.

### The stage of preventive and curate measures

Further prevention methods differed significantly. While COVID-19 containment was for all people, AIDS containment was for specific groups already prone to discrimination. For COVID-19, social distancing, handwashing and a facemask were put in place for all, although specific countries did not implement all these measures at the same time. With the arrival of the vaccines, some countries abandoned either the facemask or social distancing, leading to COVID-19 variants spreading quickly in the population. The incomplete information that being vaccinated prevents infection definitely contributed to the 4^th^ wave of COVID-19. In the case of AIDS, the main prevention measures were safe sex (for all) and clean needles (for drug users). For safe sex, many countries adopted campaigns to use condoms, although some religious countries were hesitant. As hard drug use is illegal in most if not all countries, access to clean needles (through syringe exchange programmes) was especially difficult to organize and had to be organized in the grey areas of the law. This gave those not belonging to these groups (homosexual men and drug users) a false sense of safety. Treatments of HIV have developed more and more and HIV infection is now more seen as a chronic disease.

### Lessons learned

So what lessons can be learned from this comparison?

1. Collaboration between countries is key. If we don’t work together, a pandemic is difficult to contain. For instance, the limited access to vaccines for COVID-19 in several countries (e.g. African countries) prolongs the pandemic. Sharing knowledge and skills to make vaccines and waiving the patents on these vaccines can only benefit the world (see our joint EUPHA-WEMOS statement on this, EUPHA—Wemos statement.pdf last accessed 1 December 2021)
2. The right information at the right time in an understandable way. It is essential, especially in times of social media, to get the facts right and to counter fake news. For this, we do not only need scientists who can communicate research, but also a health literate population. Both need our attention.
3. Keep health emergency preparedness plans updated! In several countries in Europe, it turned out that these plans had not been updated to the latest information. This slowed down the response to the pandemic. The newly installed HERA—Health Emergency Preparedness and Response Authority—of the European Union should improve this, as HERA’s objective is to anticipate threats and potential health crisis, through intelligence gathering and building the necessary response capacities.
4. Be open and transparent. It is crucial that we do not make up stories and facts, we need to be open and transparent not only about our certainties but also at our uncertainties.

My wish for 2022 is that COVID-19 becomes a virus similar to flu. You can get vaccinated and—if you get infected—the symptoms are mild and treatable.

## We need to apply the same urgency to fighting cancer that we are to COVID-19

SmelovVitalyLassiera LosadaMariaCorbexMarilysFerreira-BorgesCarinaKlugeHans Henri P

The pandemic is providing new perspectives and countless lessons on battling a life-threatening disease. Undoubtedly, COVID-19 has been a shock to the system, but it has also opened several windows of opportunity. Perhaps most importantly, it has shown us what we’re capable of, when united and faced with a common threat.

What we now need is to apply the same urgency to fighting cancer. Cancer is one of the major causes of death and morbidity in the WHO European Region, and accounts for more than 20% of deaths according to WHO estimates^1^.

We have all watched as COVID-19 disrupted cancer services across the European Region and the world, affecting millions of people.^2^,^3^,^4^–^5^

The pandemic has delayed cancer diagnosis and care and created service backlogs that will take months—possibly years—to address. Data suggests we could face a ‘cancer pandemic’ in the years ahead; the result of so many people diagnosed with delay or missed screening over the past two years, causing them to suffer forms of the diseases more difficult and more expensive to treat.^6^^,^^7^

One of the key recommendations of the Pan-European Commission on Health and Sustainable Development, which was tasked by the WHO Regional Office for Europe with rethinking policy priorities in light of pandemics, is that we need to act at all levels of society to heal the divisions laid bare by COVID-19.^8^

This means it is time to make the mantra of “building back better” a reality and address the long-standing, deep-seated roots of inequality.

And nowhere is this inequality clearer than in cancer diagnosis, treatment and survival.

The cancer survival rate continues to be a compelling example of inequity in health care across the Region. The difference in mortality rates across the continent for breast, cervical and lung cancers are twofold, fivefold, and tenfold, respectively.^9^

In addition, in too many countries, differential access to cancer services and out-of-pocket expenditures are pushing patients and their families into financial difficulty or near bankruptcy. This underscores the urgency of Universal Health Coverage and the need for financial protection. For many cancer patients and their families, irrespective of where they live, these are not just words. Protection from financial risk is a matter of life and death.

In the 53 countries of the WHO European Region, 30-40% of cancers are preventable, 4.8 million people are diagnosed with cancer every year of which 2.2 million people sadly lose their lives. In fact, 1 in 4 people will get a cancer diagnosis in their lifetime, and 43% of them will die from cancer.^10^

If current trends continue, the global incidence of all cancers combined will double by 2070.^11^ And we will also see growing geographical and socioeconomic inequality if measures are not taken to protect those at greatest risk.

Investing in health systems and health education is more urgent now than ever.

Addressing catastrophic consequences of inaction in cancer prevention is equally vital. And so is strengthening or even reforming health systems to bridge foundational gaps in cancer prevention, early detection, treatment and supportive care.

In short, we all have work to do, and WHO has recently scaled up its efforts.

WHO assists countries in developing and updating National Cancer Control Plans that cover the entire cancer continuum, from prevention to palliative and supportive care. Our goal is threefold:

1. A reduction in cancer incidence and mortality, improved quality of life for patients, and better use of resources.
2. Provision of state-of-the-art cancer data to shape policy and action—and stronger national cancer registries, working together with WHO’s International Agency for Research on Cancer.
3. The development of country-specific technical recommendations through assessing barriers to early detection—of particular importance in a post-COVID-19 era.

On World Cancer Day last year, as part of the European Programme of Work, we launched a pan-European cancer movement, United Action Against Cancer, with the long-term goal of eliminating cancer as a life-threatening disease. The movement brings together WHO expertise and resources, as well as relevant stakeholders (including other UN agencies) to achieve impact at country level.

This movement enhances collaboration, unites partners and reinforces political leadership. It galvanizes young people and civil society. It facilitates fast-track solutions aligned with global initiatives on cervical, breast and childhood cancers. In addition, it provides cost-effective, evidence-based policies for countries to implement effective prevention measures, and quality screening and management of cancer cases. And finally, it enables policymakers to make the right decisions to tackle cancer effectively and faster.

With this initiative, we are promoting evidence-based screening, maximizing benefits, minimizing harms; tackling inequality, and fighting inadequate cancer policies often influenced by commercial determinants, lacking in scientific evidence.

WHO’s work is guided by the Sustainable Development Goals. Cancer control is a key priority if we are to reach the target of reducing premature mortality from noncommunicable diseases by 2030. We believe that together, it is achievable, if we fight cancer with the same urgency as COVID-19.

## 15th European Public Health Conference, 9–12 November 2022, Berlin, Germany

BusseReinhard
Chair 15th EPH Conference 2022
BarnhoornFloris
EPH Conference Office

### Welcome to Berlin 2022

The 15th EPH Conference in Berlin will be the first conference since Marseille 2019 to take place live and in person! The spectacular Hub27 venue provides us with ample opportunities for informal chatting, personal exchange and live networking. We hope you will join us and we truly look forward to seeing everyone!

The focus of Berlin 2022 will be on health systems. We want to explore ways in which the public health community can rally around the WHO health system framework to bring about better population health. We need to accept the fact that inequity in health has taken on new dimensions and now more than ever, poses a threat with global ramifications. We have learned from the Covid-19 pandemic that infectious diseases know no boundaries. We have seen the successful development of vaccines but also the spread of the Delta variant around the globe as these vaccines have not yet been available to everyone. The conclusion is simple: the EU must take on a more active role in global health. This includes taking a stand on expansive issues such as planetary health, climate change, environmental degradation, water and food security, migration, gender, and poverty. More tangibly, it means we must fight to increase global health equity. Europe must use its knowledge and influence to balance out market forces, expand regulatory oversight and create socially responsible systems for health, especially when it comes to the distribution of vaccines and essential medicines.

Digitalization is a key area where recent developments require us to exchange ideas and come up with new approaches. The past decades have seen significant developments in IT processing capacity as well as health data collection and eHealth applications. We need to ensure that the vast amounts of data generated are well protected on the one hand, while also being available and usable for public health interventions. We have devoted one subtheme of the conference to “Harmonizing information systems and health reporting”. We also need to train more of our public health workforce to be able to work with data effectively. Knowing who is sick when, where and why is of critical importance if we wish to successfully combat emerging health threats, disease outbreaks and future epidemics.

We want the discussions at the 15th EPH Conference to result in a Europe characterized by resilient health systems, universal health coverage (UHC), a well-trained and highly qualified clinical and public health workforce, and governance structures that take a stand and shape health systems globally. We look forward to seeing you in Berlin!

### Abstract submission for Berlin 2022

Participate at Berlin 2022 by sharing your work with colleagues. The EPH Conference offers excellent opportunities to learn from the latest research and practice, to network with experts and colleagues and to expand your professional horizons. Abstracts are invited for workshops and single presentations in all areas of public health research, practice, policy and education. Abstract submission is open from 1 February to 1 May 2022. Abstracts are considered for workshops, oral, pitch and poster presentations. Do not miss the opportunity to share your work at the largest public health event in Europe. For less experienced abstract submitters a Mentor programme is set-up. Be recognised for your hard work. Detailed instructions can be found on www.ephconference.eu.

**Themes of the 15th EPH Conference:**

- Preparing for the unexpected: lessons learned from Covid-19 and other disasters
- Expanding horizons: the role of Europe in global health
- Achieving universal health coverage (UHC): reducing health inequity
- Harmonizing information systems and health reporting: ensuring function and use
- Measuring health system performance: using data to improve population health
- Sustaining high quality care: interprofessional training for our clinical and public health workforce
- Utilizing the transformational potential of health promotion: accelerating action on SDGs
